# Supplementary material for: Gene regulatory network reconstruction using single-cell RNA sequencing of barcoded genotypes in diverse environments
Source: eLife. 2020 Jan 27;9:e51254. doi: 10.7554/eLife.51254 (PMC7004572; doi:10.7554/eLife.51254)
Supplement: Source code 5. — The R markdown file to create this document is contained in Source code 1. [file elife-51254-code5.zip › Jackson_2019_Supplemental_Data_6.html]

 

 

 

 
 
 


 


 Jackson et al eLife (2019) Figures 

 
 
 
 
 
 
 
 
 
 
 

 
 
 


 


 

 

 


 

 
 

 


 


 Jackson et al eLife (2019) Figures 
  Chris Jackson  
  August 3, 2019  

 


 
 Figure 2 
   
  Figure 2: Gene expression of single Yeast Cells Cluster Based on Environmental Growth Condition (A)  Normalized density histograms of raw UMI counts of the core glycolytic genes ENO2 and PDC1, and the alcohol respiration gene ADH2 in each environmental growth condition. Mean UMI count for each of the 12 different strain genotypes within each growth condition are plotted as dots on the X axis. (B-C) Uniform Manifold Approximation and Projection (UMAP) projection of log-transformed and batch-normalized scRNAseq data. Axes are dimensionless variables V1 and V2 with arbitrary units, here omitted. Individual cells are colored by environmental growth condition (B) or by strain genotype (C). Growth conditions are abbreviated as in Table 1. 

 
 
 Figure 3 
   
  Figure 3: Cells Within Conditions Cluster According to Cell Cycle Genes  (A) Cells from each growth condition were separately normalized and transformed into 2-dimensional space using UMAP. The log-transformed, normalized expression for each cell of (i) the G1-phase specific marker PIR1, (ii) the G1-phase daughter-cell specific marker DSE2, (iii) the S-phase specific marker histone 2B (HTB) is shown; (iv) the genotype and (v) the cluster membership of each cell. (B) Summary of clustered single cell expression within the YPD and RAPA growth conditions (i) Proportion of cells from a specific strain genotype within each cluster (ii) The mean log-transformed, normalized expression of the G1- and S-phase marker genes, as well as a hexokinase gene HXK2 for each cluster (C) Schematic of the mitotic cell cycle with expression of DSE2, PIR1, and HTB genes annotated. 

 
 
 Figure 4 
   
  Figure 4: Impact of Deleting Transcription Factors on Gene Expression  (A) Violin plots of the log2 batch-normalized expression of the general amino acid permease gene GAP1 in YPD, RAPA, ammonium-limited media, and urea-limited media. (B) Count of differentially expressed genes in each combination of growth condition and strain genotype. Data were transformed to pseudobulk values by summing all counts for each the six biological replicates for each genotype and then analyzed for differential gene expression using DESeq2 [1.5-fold change; p.adj < 0.05]. (C) Log2(fold change) of genes differentially expressed in TF knockout strains compared to wildtype, when grown in YPD. Asterisks denote statistically significant differences in gene expression [1.5-fold change; p.adj < 0.05]. 

 
 
 Figure 5 
   
  Figure 5: Model Performance and Impact of Data Imputation, Prior Selection, and Multitask learning on Network Inference using the Inferelator  (A) Model performance of Inferelator (TFA-BBSR) network inference after shuffling priors [Neg. Shuffled], on a simulated negative data set [Neg. Data], on the unaltered count matrix [No Imputation], and after imputing missing data from the count matrix using the MAGIC, ScImpute, and VIPER packages. Model performance is shown using area under the precision-recall curve [AUPR], as well as the number of network edges using a precision (> 0.5) cutoff, and the number of network edges using a confidence (> 0.95) cutoff. Each point plotted in gray is a separate cross-validation analysis, with mean +/- one standard deviation plotted in black (n=10). (B) Median AUPR after cross-validation (n=10) and resampling to different numbers of cells, for priors extracted from the gold standard [GS], the YEASTRACT database, Bussemaker et al, priors predicted from ATAC-seq data and motif searching, and no prior data. (C) AUPR of separate cross-validation network inference using cells from all growth conditions, or from individual conditions separately. Each cross-validation (n=10) was downsampled to the same number of cells. (D) Cross-validation (n=10) using the YEASTRACT prior data. Networks are learned for all conditions together [BBSR (ALL) ?], for all conditions individually with TFA-BBSR followed by combination [BBSR (BY TASK) ?], and multi-task learning all tasks are learned together and then combined [AMuSR (MTL) ?]. Models are evaluated by (i) AUPR on the aggregate, final network and (ii) AUPR for each task-specific subnetwork from BBSR (BY TASK) (?) and AMuSR (MTL) (?). 

 
 
 Figure 6 
   
  Figure 6: Reconstruction of a Gene Regulatory Network Identifies New Regulatory Relationships  A network inferred from the single-cell expression data using multi-task learning and the YEASTRACT TF-gene interaction prior, with a cutoff at precision > 0.5. (A) Network graph with known interaction edges from the prior in gray and new inferred interaction edges in red (B) Network graph of the 11 nitrogen-responsive transcription factors with known edges from the prior in gray and new edges in red (C) The number of interactions for each TF; interaction edges present in the prior that are not in the final network are included in black. The nitrogen TFs knocked out in this work are labeled in blue, and TFs with gene ontology annotations for mitotic cell cycle are annotated in green (D) Gene ontology classification of network interactions by the GO slim biological process terms annotated for the target gene and the regulatory TF (the GO term transcription from RNA pol II is omitted from the annotations for regulatory TFs). 

 
 
 Figure 7 
     
  Figure 7: Coordinated regulation of Nitrogen Response and Cell Cycle  (A) A gene regulatory network showing target genes that are regulated by at least one nitrogen TF (blue) and at least one cell cycle TF (green). Target gene nodes are colored by GO slim term. Newly inferred regulatory edges are red and known regulatory edges from the prior are in gray. Transcription factor activity (TFA) is calculated from the learned network and then scaled to a z-score over all cells which do not have that TF deleted (e.g. gcn4? cells are omitted from the calculation for GCN4 TFA). The mean TFA z-score for four selected conditions is inset for GAAC and NCR TFs (B) TFA for cell cycle TFs for each cell in the YPD growth condition. 

 
 
 Supplemental Figure 2-1 
   
  Supplemental Figure 2-1: Quality Control of Single-Cell RNA Sequencing Data  (A) The number of cells that pass all quality control and preprocessing filters for each growth condition. Each genotype has multiple independently barcoded biological replicates that are plotted separately within each condition. The mean number of cells for each genotype within a condition is plotted as a horizontal line. (B) The mean count of unique transcripts, determined by UMI, for each growth condition. Biological replicates are plotted separately for each genotype within each condition. The mean number of transcripts per genotype is plotted as a horizontal line. (C) The distribution of unique transcript count per cell across all growth conditions. (D) The distribution of unique transcript counts per cell across all genotypes. 

 
 
 Supplemental Figure 2-2 
   
  Supplemental Figure 2-2: Single-Cell RNA Expression Comparison  (A) Pairwise ranked gene expression plots of cells grown in YPD to mid-log phase. Data sets are bulk counts [TRIZOL] from FY4/FY5 diploids (n=6), 10x-based 3’ end-labeled single-cell counts [10x-scRNA] from FY4/FY5 diploids (n=976), 5’ end-labeled single-cell counts [yscRNA (2019)] from BY4741 haploids (n=127), SCnorm-calculated normalized counts [Gasch (2017)] from BY4741 haploids (n=163), and bulk transcripts per kilobase million [GSE135430] from BY4741 haploids (n=12). (B) Correlation heatmap showing spearman’s rank correlation between each sample. (C) Counts after de-artifacting with UMIs for the bulk experiment on RNA extracted with TRIZOL. 

 
 
 Supplemental Figure 2-3 
   
  Supplemental Figure 2-3: Expression of Categories of Genes in Single Cells  UMAP projection of log-transformed and batch-normalized scRNAseq data, colored by: (A) Condition, as Figure 2B (B) Total raw count of transcripts (C) Percentage of transcripts which are ribosomal genes (D) Percentage of transcripts which are ribosomal biogenesis genes (E) Percentage of transcripts which are induced environmental stress response (iESR) genes (F) Percentage of transcripts which are mitochondrially-encoded genes 

 
 
 Supplemental Figure 2-4 
   
  Supplemental Figure 2-4: Measures of Gene Variance in Each Condition  (A) Coefficient of variation (standard deviation over mean) plotted against mean of each gene for each growth condition. Both axes are plotted on a log scale. (B) The mean pearson residuals (residuals over expected standard deviation) of a regularized negative binomial regression model calculated for each gene by the R package sctransform. The mean gene expression is plotted on a log scale. 

 
 
 Supplemental Figure 3-1 
   
  Supplemental Figure 3-1: Expression of Important Genes For Clustering  (A) Gene expression heatmap of genes that are specific for clusters of cells in YPD. Genes name are colored green for G1 phase genes, yellow for S phase genes, purple for G2 phase genes, and blue for M phase genes (B) Summary of clustered single cells within growth conditions (i) Proportion of cells within each cluster that consist of a specific strain genotype (ii) The mean within each cluster of log-transformed, normalized expression of the G1- and S-phase marker genes, as well as a hexokinase gene HXK2 that is unlikely to be responding to cell cycle 

 
 
 Supplemental Figure 3-2 
   
  Supplemental Figure 3-2: Some Conditions Have Stress Response Clusters  Cells from each growth condition were separately normalized and transformed into 2-dimensional space using UMAP. These single cells are colored by (A) Total raw UMI count (B) Percentage of transcripts which are ribosomal genes (C) Percentage of transcripts which are induced environmental stress response (iESR) genes (D) Percentage of transcripts which annotated as G1 phase genes (E) Percentage of genes which are annotated as S phase genes (F) Percentage of genes which are annotated as G2 phase genes (G) Percentage of genes which are annotated as M phase genes 

 
 
 Supplemental Figure 4 
   
 Supplemental Figure 4: Differential Gene Expression Varies by Condition** (A) Distribution by strain genotype of the log2 batch-normalized expression of the ammonium permease gene MEP2 and the glutamine synthetase gene GLN1 in YPD, RAPA, and ammonium limitation. (B) Number of differentially expressed genes identified after bulking between wild-type cells in each growth condition (C) Log2(fold change) of genes differentially expressed in TF knockout strains compared to wildtype, when grown in YPD and then treated with rapamycin. Asterisks denote statistically significant differences in gene expression [1.5-fold change; p.adj < 0.05]. 

 
 
 Supplemental Figure 5 
   
  Supplemental Figure 5: Low-Dimensional Clustering of Imputed Data  Scatter plot after UMAP into 2-dimensional space. Unmodified data (A) is compared to imputation methods to recover missing data using MAGIC (B), ScImpute (C), and VIPER (D). 

 
 
 Supplemental Figure 6 
   
  Supplemental Figure 6. Summary of Learned GRN  (A) Histogram of the number of regulators per target gene in the learned and prior network (B) Hexagonal heatmap of the ranked expression of a gene against the number of regulators for that gene; r2 is calculated using Spearman’s Rank correlation (C) The number of interactions for a TF that are activating (positive) or repressing (negative). Bars are colored by the number of separate conditions in which the interaction is identified, with condition-specific networks selected using a precision threshold of 0.5 (D) The number of unique TF-gene interactions identified in condition-specific networks (E) The distribution of learned and prior interactions from the final, aggregate network in condition-specific networks (F) Evidence for learned network edges which are not in the prior. Interactions where YEASTRACT has evidence for a change in gene expression when the TF is perturbed are in tan. Interactions where YEASTRACT has evidence that the TF physically localizes to the gene are in blue. Interactions where YEASTRACT has no annotated evidence are in red. 

 
 
 Supplemental Figure 7 
   
  Supplemental Figure 7. Cell Cycle TF Activity Clusters within Growth Conditions  Cells grown in YPD are plotted after UMAP with (A-B) z-Score of the calculated transcription factor activity (TFA) based on the learned network for (A) select nitrogen TFs and (B) cell cycle TFs (C) Log2 of the expression of cell cycle TFs (D) Log2 of the expression of cell cycle target genes 
 


 

 

 
 

 
 
